# The Role of Lattice Matching Techniques in the Characterization of Polymorphic Forms

**DOI:** 10.6028/jres.116.007

**Published:** 2011-04-01

**Authors:** Alan D. Mighell

**Affiliations:** National Institute of Standards and Technology, Gaithersburg, MD 20899-0001

**Keywords:** derivative lattice analysis, lattice-matching techniques, matrix strategy, polymorph characterization, pseudo-polymorphs, reduced cell strategy

## Abstract

An inspection of the recent literature reveals that polymorphism is a frequently encountered phenomenon. The recognition of polymorphic forms plays a vital role in the materials sciences because such structures are characterized by different crystal packing and accordingly have different physical properties. In the pharmaceutical industry, recognition of polymorphic forms can be critical for, in certain cases, a polymorphic form of a drug may be an ineffective therapeutic agent due to its unfavorable physical properties. A check of the recent literature has revealed that in some cases new polymorphic forms are not recognized. In other instances, a supposedly new polymeric form is actually the result of an incorrect structure determination. Fortunately, lattice-matching techniques, which have proved invaluable in the identification and characterization of crystal structures, represent a powerful tool for analyzing polymorphic forms. These lattice-matching methods are based on either of two strategies: (a) the *reduced cell strategy*–the matching of reduced cells of the respective lattices or (b) the *matrix strategy*–the determination of a matrix or matrices relating the two lattices coupled with an analysis of the matrix elements. Herein, these techniques are applied to three typical cases–(a) the identification of a new polymorphic form, (b) the demonstration that a substance may not be a new polymorphic form due to missed symmetry, and (c) the evaluation of pseudo polymorphism because of a missed lattice. To identify new polymorphic forms and to prevent errors, it is recommended that these lattice matching techniques become an integral part of the editorial review process of crystallography journals.

## 1. Introduction

The recognition of polymorphic forms plays a vital role in the materials sciences because such structures are characterized by a different crystal packing and accordingly have different physical properties. As research using the Cambridge Structural Database (CSD) [1] has demonstrated, polymorphic forms of crystalline structures are often encountered. Many studies on packing and on polymorphic forms have been carried out using crystallographic structural data in the CSD [2–4]. Likewise a survey of recent publications in Acta Crystallographica shows that it is not uncommon for a new compound [5] to be a polymorphic form of an extant compound [6].

For pharmaceutical compounds the phenomenon is very common [7]. Recently, Grzesiak et al. [8] published a “comparison of the four anhydrous polymorphs of carbamazepine.” In the pharmaceutical industry, such recognition of polymorphic forms can be critical for, in certain cases, a polymorphic form of a drug may be an ineffective therapeutic agent due to its unfavorable physical properties. Because of their importance, it is necessary to determine if two compounds are actually polymorphs.

### 1.1 Not All Structures Said to be Polymorphs are Polymorphs

Two substances with the same formula but crystallizing in structures based on different lattices are generally considered polymorphs. But not all compounds reported as polymorphs are actually polymorphs. In some cases, they may not be because of an error in structure solution—one determined structure is correct whereas the presumptive polymorphic form is derived from an incorrect structure solution. For example, Clemente and Marzotto [9] recently published a paper with 30 space-group corrections including two examples of false polymorphism. Karami et al. [10] in a recent paper on “further errors in polymorph identification" showed that by taking insufficient data one can obtain a false polymorph. In a similar vein, Marsh [11] demonstrates that many structures initially described in monoclinic space group *Cc* are “better described in higher symmetries.” Many of these incorrect crystal structures result from an over reliance on highly automated structure-determination methods, a failure to understand the limits of these procedures, and a lack of scrutiny of the results. Fortunately, lattice analysis methods can play a vital role in polymorph characterization and identification.

## 2. The Role of Lattice-Matching Techniques in Polymorph Characterization

By focusing on the lattice, the investigator can deduce if two structures with the same formula are polymorphs—(a) if the lattices are the same, the structures are almost always identical, and (b) if the lattices are different, the structures are polymorphs. To determine if the two lattices are the same or different, the investigator can use lattice-matching techniques [12–18]. These lattice-matching methods are based on either of two strategies: (a) the *reduced cell strategy*—the matching of reduced cells of the respective lattices or (b) the *matrix strategy*—the determination of a matrix or matrices relating the two lattices and analyzing the matrix elements.

Lattice matching procedures have proved to be powerful tools in identification and in data evaluation procedures. Extensive experience with large crystallographic databases [1, 19, 20] has demonstrated that if one obtains a lattice match for two structures with the same formula, then the structures are almost always identical. Furthermore, these lattice identification methods are robust and if properly applied work well in spite of certain types of experimental error. For example, it is not uncommon to encounter a pair of cases in which the lattice of one solved structure is correct whereas the other corresponds to a derivative lattice of the same compound. Nevertheless, it is still possible to obtain a lattice match by using the derivative lattice procedure as outlined in Fig. 1 of Ref. [12].

## 3. Discussion

Herein, we analyze three cases published in the recent literature where polymorphism plays an essential role. Lattice methods are used to distinguish whether two crystallographic structures with the same formula represent polymorphic or pseudo- polymorphic forms. In the first case, the structures are shown to be polymorphic forms. The second and third cases demonstrate how errors in symmetry and lattice determination, respectively, can lead to pseudo-polymorphic forms.

### 3.1 Case I: Reduction Confirms Actual Polymorphic Forms—a Tetrapyrazole Complex and Carbamazepine as Examples

#### 3.1.1 Two Polymorphic Forms of a Tetrapyrazole Complex—Ni(NCS)_2_(C_3_H_4_N_2_)_4_

Recently two crystal structure determinations for a tetra pyrazole complex—Ni(NCS)_2_(C_3_H_4_N_2_)_4_ were reported in the scientific literature. The first was published by Takahashi et al. in 2006 [6] and the second by Yan in 2007 [5]. Although the formulas are the same, the second publication did not reference the first. Do these two independent publications describe polymorphic forms or are the two structures identical?

Lattice-analysis methods (e.g., cell reduction) can be employed to resolve problems of this type. For each structure, the author’s original unit cell was reduced. As the data in Table 1 reveal, the two reduced cells are clearly different. Therefore, the crystal structures for the two compounds are polymorphic forms.

In this case, the authors’ original cells (Table 1) can be directly compared as they are conventional cells that are based on the shortest vectors in the *ac* plane. Such a comparison also reveals that the two crystal structures are different. Note that both the *b*-axes [10.863(2) vs. 12.3994(5)Å)] and the *β* angles [117.485(2) vs. 105.341(5)°] are clearly different. (See Table 2 in Ref. [14] for transformation matrices from reduced to conventional cells).

Many related complexes of the general formula Me (Pz_4_) X_2_ (where Me = transition metal, Pz = pyrazole and X = a halide, SCN^–^ or pseudo halide) have been reported in the literature but usually in only one crystalline form. This thiocyanate complex (Table 1) is a particularly interesting case as it crystallizes in two forms—one of the polymorphs is related to one series of isostructural compounds and the other is linked to a second series of related complexes. For example, the structure of the tetrapyrazole manganese NCS complex published by Lumme et al. (1983) [22] is similar to the structure of Takahashi [6]. On the other hand, the lattice of the tetrapyrazole Mn Br complex published by Ruth et al. [23] is similar to the complex of Yan [5].

#### 3.1.2 The Four Polymorphic Forms of Carbamazepine—C_15_H_12_N_2_ O

Carbamazepine, an anticonvulsant, is a widely studied compound in the pharmaceutical industry. Because of its propensity to crystallize in different structures, this compound has played an important role as a reference compound in polymorphic studies.

Crystal structures of four polymorphic forms of carbamazepine have been reported in the literature [8]. The lattice data for the four forms are summarized in Table 2. For each form, we give the original cell reported by each author and the corresponding reduced cell. By comparing the standard reduced cells, it is confirmed that the compounds are polymorphs. Note that for form III, two different original cells—one conventional [25] and the other unconventional [26]—were reported.

Compounds closely related to carbamazepine are also known to crystallize in polymorphic forms. For example, four polymorphic forms have been reported for 10, 11-dihydrocarbamazepine [28]. Again a comparison of the standard reduced cells shows that the crystal lattices are different and the forms unique.

### 3.2 Case II: Questionable 2nd Polymorphic Form Due to Lattice/Formula Match With Existing Compound—Triphenylphosphine oxide hemi-hydrate (TPPO • 0.5H_2_O) as an Example

Recently, Ng (2009) [29] reported a “second mono-clinic modification” for triphenylphosphine oxide hemihydrate, C_18_H_15_ OP • 0.5H_2_O. However, the lattice for this phase bears a strikingly close resemblance to the compound (orthorhombic *F*) previously reported by Baures and Silverton (1990) [30]. Using NIST*LATTICE [18], it was established that the reduced cells (Table 3) for these two compounds are very close. Experience in data evaluation has shown that two phases are usually identical when the reduced cells and the formulas are the same [12].

For the second monoclinic modification, it is further noted that the metric symmetry (Table 4) exceeds the crystal symmetry—i.e., the metric symmetry is *F*-centered orthorhombic whereas the crystal symmetry is reported as *C*-centered monoclinic (Space Group *Cc*). This is a warning flag. In fact, Marsh [11] in the “Perils of *Cc* revisited” cites 98 “structures originally described in space group *Cc* which are better described in higher symmetries.” Fifteen of these structures represent cases for which the structure is best described in the orthorhombic space group *Fdd*2. The structure of triphenylphosphine oxide hemihydrate is a similar example. In fact, the reduced cell for C_18_H_15_ OP • 0.5H_2_O of Ng [29] can be transformed by the matrix (1 −2 0 / −1 0 2 / −1 0 0) to an orthorhombic *F*-centered cell similar to that reported by Baures and Silverton (1990) [30]. Thus, it is concluded that the Ng [29] and Baures & Silverton [30] crystal structures are very similar or identical. To prove the identity, a successful refinement of the second monoclinic modification in the orthorhombic space group *Fdd*2 would be required.

As both of the above structures were published in Acta Crystallographica, it is surprising that the journal’s automated evaluation procedures did not alert the editors or the author to a potential identity problem. In this case, a warning—that there is a lattice/formula match [12] for the two structures and that the metric symmetry exceeds crystal symmetry for the second structure—would have been helpful.

### 3.3 Case III: False 2nd Polymorphic Form Due to Missed Lattice—Furosemide as an Example

#### 3.3.1 Experimental Errors in Data Collection Lead to False Polymorphic Forms

Sometimes a presumptive second polymorphic form of a compound results from the fact that the structure determination was based on an incomplete set of diffraction data. At first glance, the traditional requirements for a correct structure determination—low R-value, reasonable bond distances, etc.,—are satisfied. However, the fact that a few atoms in the molecule are disordered serves as an indicator of a potential error in the structure determination. Karami et al. [10] clearly demonstrate the problem in their informative manuscript entitled “Further errors in polymorphic identification: furosemide and finasteride.” The authors state that a “reassessment of the reported single-crystal x-ray diffraction characterization of polymorphs of furosemide and finasteride shows that, in each case, incomplete data collections have resulted in the mistaken identification of two forms that are, in fact, identical.”

In practice, an error in a structure determination can occur because certain structures produce a subset of diffraction data consisting of weak reflections. By missing such a category of weak reflections, the experimentalist will inadvertently determine a derivative sublattice rather than the correct lattice. When the structure is solved on the basis of a derivative lattice, one may obtain what appears, in most respects, to be a reasonable structural solution—except for some disorder as noted above. However, once the structure is solved with all the diffraction data, the disorder will disappear. It is not uncommon for both the “subcell structure” and the correct structure to be reported in the literature masquerading as polymorphic forms. Such is the case for furosemide [10] where the correct structure [31] as well as two incorrect structures [32, 33] based on sublattices were reported in the scientific literature. Lattice methods—such as the reduced cell, derivative cell, and matrix strategies—are powerful tools to recognize and solve problems of this type.

##### 3.3.1.1 The Reduced-Derivative-Cell Strategy Reveals Pseudo Polymorphism

In Table 5, a lattice analysis of this case of pseudo polymorphism is given. In the Table, we summarize the lattice data—author’s original cells and calculated reduced cells—for the three separate determinations of furosemide [31–33] reported in the scientific literature. Inspection of the data in the table reveals that structures 2 and 3 are the same as their reduced cells are identical. This was noted by Karami et al. [10] but they went on to state that there was no obvious *reduced cell link* with structure 1. However, this vital link can be clearly established via the matching of the reduced cell of an appropriate derivative lattice. As a derivative subcell (reduced) of structure 1 matches the reduced cells for structures 2 and 3, it is concluded that the three structures are actually the same.

##### 3.3.1.2 The Matrix Strategy Reveals Compound Identity

Alternatively, the *matrix strategy* can be used to establish the identity of the three structures. This is shown in Table 6. Using NIST*LATTICE [18], the matrices relating the unit cells for the structures were calculated. As the matrix relating structures 2 and 3 consists of integer elements with a determinant of 1, one concludes that lattices of 2 and 3 are identical. As the table shows, the transformation matrix relating 2 → 1 (or 3 → 1) has integer elements and a determinant of 2. Therefore the unit cells for structures 2 and 3 are subcells of the unit cell for structure 1.

#### 3.3.2 Confirmation of Form I and the Discovery of Two New Polymorphic Forms (II & III) of Furosemide

Recently, Babu et al. [34] further confirmed—via a redetermination—that the Lamotte et al. [31] structure of Furosemide is correct and designated it as Form I. In addition, they reported two new Forms (II & III) for Furosemide. The uniqueness of all three forms was confirmed using lattice matching techniques. The *reduced cell strategy*—the standard reduced cells for all three forms were found to be different—and the *matrix strategy* both revealed that the lattices for the three forms are distinct.

## 4. Conclusion

The Lattice based methods—reduced cell, derivative lattice, and matrix analysis—represent an invaluable and powerful tool for the identification and characterization of polymorphic forms. They are very straightforward, easy to apply, and reliable. They can be applied at an early stage in the analysis—as soon as the unit cells for the structures have been determined. These methods allow the investigator to distinguish whether two (or more) structures with the same formula are polymorphs or pseudo polymorphs. If the reduced cells for two crystal structures are the same, then the structures are almost always the same. When there is not a match of a reduced cell for original (or a derivative lattice) lattices then the compounds represent polymorphic forms. Alternatively, one can calculate the matrix relating two lattices. An analysis of the matrix elements will reveal the lattice relationship.

These lattice procedures can readily be automated and should be an integral element of an editorial review process for crystallography journals. As the editorial procedures for Acta Crystallographica Sec. E are already heavily automated—required to meet the largest throughput of crystal structures in the scientific literature—this would be the ideal place to start. A newly determined structure could routinely be checked against all extant structures with the same formula to ascertain if it is the same structure or a polymorphic form. This check can be carried out via lattice/formula matching techniques [12–18]. Today such a check is feasible as the required reduced cell file to match against can be readily produced from the existing large and comprehensive crystallographic databases—e.g. the Cambridge Structural Database (CSD) [1] and the Inorganic Crystal Structure Database (ICSD) [19]. In addition to identifying polymorphic forms, such a routine check would assist in preventing the inadvertent publication of duplicate structures and in establishing structural relationships.

## Figures and Tables

**Table 1:** Application of the *Reduced Cell Strategy* to evaluate two reported structures of Tetrakis(1*H*-pyrazole-κ*N*^2^) bis (thiocyanato-κN) nickel(II)—[Ni(NCS)_2_(C_3_H_4_N_2_)_4_]. As the formulas are the same but the reduced cells different, the structures are clearly polymorphic forms

	Structure 1		Structure 2	
	Original Cell	Reduced Cell^c^	Original Cell	Reduced Cell
*a* (Å)^a^	14.046(3) b	8.878	11.4327(7)	8.433
*b*	10.863(2)	8.878	12.3994(5)	8.433
*c*	14.862(3)	14.260	14.2802(8)	14.280
α(º)	90.0	102.94	90.0	100.33
β	117.485(2)	104.01	105.341(5)	100.33
γ	90.0	104.56	90.0	94.65
*V*(Å^3^)	2011.6(7)	1005.9	1952.2(2)	976.1
*SG*	*C*2 / *c*	*P*	*C*2 / *c*^d^	*P*
*Z*	4	2	4	2
*Ref*	Yan (2007) [5]		Takahashi et (2006) [6]	

a The labels in this column correspond to the unit cell edges & angles (*a*, *b*, *c*, α, β, γ), the unit cell volume (*V*), the space group (*SG*), the molecules/unit cell (*Z*), and the reference (*Ref*).

b The numbers in parentheses corresponds to the estimated standard deviations.

c The reduced cells given in the Table were calculated using NIST*LATTICE [18].

d Original space group corrected [21].

**Table 2:** Carbamazepine (C_15_H_12_N_2_O)—Four polymorphic forms. A comparison of the standard reduced cells shows that the four crystal lattices are clearly different and the forms are unique

	Form I	Form II	Form III^a^	Form III	Form IV
Authors’ original cells					

*a* (Å)^b^	5.1705(6)^c^	35.454(3)	7.537(1)	7.529(1)	26.609
*b*	20.574(2)	35.454(3)	11.156(2)	11.148(2)	6.9269
*c*	22.245(2)	5.253(1)	13.912(3)	15.470(2)	13.957
α(º)	84.12(4)	90	90	90	90
β	88.01(4)	90	92.86(2)	116.17(1)	109.702
γ	85.19(4)	120	90	90	90
*V*(Å^3^)	2344.8(5)	5718(1)	1168.3	1165.3	2421.9
*Sys*	Triclinic	Trigonal	Monoclinic	Monoclinic	Monoclinic
*SG*	$P\bar{1}$	$R\bar{3}$	*P*2_1_/*n*	*P*2_1_/c	*C*2/*c*
*Z*	8	18	4	4	8
*D_x_* g/cm^3^	1.339	1.235	1.343	1.35	1.296

Reduced cells					

*a* (Å)	5.170	5.253	7.537	7.529	6.927
*b*	20.574	20.544	11.156	11.148	13.748
*c*	22.245	20.544	13.912	13.902	13.957
α(º)	84.12	119.28	90	90	109.04
β	88.01	94.89	92.86	92.91	90
γ	85.19	94.89	90	90	104.59
*V*(Å^3^)	2344.8	1906.1	1168.3	1165.3	1211.0
*Lat*	P	P	P	P	p
*Ref*	Grzesiak et al. (2003)[8]	Lowes et al. (1987)[24]	Himes et al. (1981)[25]	Reboul et al. (1981)[26]	Lang et al. (2002)[27]

a Two independent structural studies were reported for Form III. This authors’ original cell is the conventional cell as it is based on the shortest vectors in the *ac* plane.

b The labels in this column—not previously defined in Table 1—correspond to the crystal system (*Sys*), the density (*D_x_*) and the lattice type (*Lat*).

c The numbers in parentheses are estimated standard deviations.

**Table 3:** Application of the *Reduced Cell Strategy* to evaluate potential identity of two reported polymorphic forms of Triphenylphosphine oxide hemihydrate, C_18_H_15_ OP • 0.5H_2_O. As the formulas are the same and the reduced cells similar, it is likely that the two structures are very close or identical

	Structure 1		Structure 2	
	Original Cell	Reduced Cell^a^	Original Cell	Reduced Cell
*a* (Å)	19.794(18)	9.459	9.4313(1)	9.431
*b*	32.540(12)	10.969	32.1930(4)	10.844
*c*	9.459(6)	16.9435	10.8435(1)	16.773
α(º)	90	83.09	90	82.99
β	90	73.79	115.742(1)	73.67
γ	90	64.46	90	64.26
*V*(Å^3^)	6092.5(22)	1523.1	2965.59(6)	1482.8
*SG*	*Fdd2*	*P*	*Cc*	*P*
*Z*	16	4	8	4
*Ref*	Baures & Silverton (1990) [30]		Ng (2009) [29]	

a The reduced cells given in the Table were calculated using NIST*Lattice [18].

**Table 4:** The reduced forms^a^ for two reported polymorphic forms of triphenylphosphine oxide hemihydrate, C_18_H_15_OP • 0.5H_2_O. The reduced form for Structure 2 is very similar to that of Structure 1. Therefore the metric symmetry of the lattice for two compounds is essentially the same —Orthorhombic *F*-centered

Reduced Form #26^b^			Structure 1 Reduced Form (Å^2^)			Structure 2 Reduced Form (Å^2^)		
***a***·***a***	***b***·***b***	***c***·***c***	89.47	120.32	287.08	88.95	117.58	281.34
***a***·***a****/*4	***a***·***a****/*2	***a***·***a****/*2	22.37	44.74	44.74	22.21	44.48	44.42
			Baures & Silverton (1990) [30]			Ng (2009) [29]		

a The reduced cells given in the Table were calculated using NIST*Lattice [18].

b Reduced form #26 corresponds to an *F*-centered orthorhombic lattice. See Ref. [14] for a detailed discussion of the reduced forms as well as for a Table with the 44 reduced forms and corresponding conventional cells.

**Table 5:** Application of the *Reduced Cell*^a^ *Strategy* to determine “polymorph” identity for Furosemide, C_12_H_11_CIN_2_O_5_S. A reduced subcell^b^ of Structure 1 is the same as the reduced cells of Structure 2 and Structure 3. This strongly indicates that the three structures are identical. The Lamotte et al. [31] original cell is correct whereas the original cells for Structure 2 and 3 are actually subcells

	Structure 1			Structure 2		Structure 3	
	Original Cell	Reduced Cell^a^	Reduced *Subcell*^c^	Original Cell	Reduced Cell	Original Cell	Reduced Cell
*a* (Å)	10.467(12)	9.584	5.234	5.251	5.251	5.234(3)	5.234
*b*	15.801(15)	10.467	8.763	8.771	8.771	8.751(6)	8.751
*c*	9.584(10)	15.725	14.988	15.038	15.038	15.948(15)	14.982
α(º)	71.87	93.47	78.10	101.77	78.23	103.68(12)	77.42
β	115.04	107.26	89.14	89.05	89.05	69.94(9)	89.10
γ	108.48	115.04	82.28	97.57	82.43	95.59(12)	84.41
*V*(Å^3^)	1332.84	1332.84	666.42	672.09	672.09	666.58	666.58
*SG*	$P\bar{1}$	*P*	*P*	$P\bar{1}$	*P*	$P\bar{1}$	*P*
*Z*	4	4	2	2	2	2	2
*Ref*	Lamotte, Campsteyn, Dupont & Vermeire (1978) [31]			Fronckowiak & Hauptman (1976) [32]		Shin & Jeon (1983) [33]	

a The reduced cells given in the Table were calculated using NIST*Lattice [18].

b Matrices to calculate the required subcells are given in Table 8 of Ref. [14].

c Transformation Matrix: Reduced Subcell → Reduced Original Cell (Structure 1) = 1 –1 0 / −2 0 0 / 0 1−1 (Δ = 2).

**Table 6:** Application of the *Matrix Strategy* to determine polymorph identity for Furosemide, C_12_H_11_CIN_2_O_5_S. The nature of the transformation matrices reveals that the authors’ cells for structures 2 and 3 are subcells of the cell for structure 1

	Structure 2 Author’s Cell^a^	Transformation Matrix (3 × 3)^b^	Structure 1 Author’s Cell	Transformation Matrix (3 × 3)^c^	Structure 3 Author’s Cell
*a*(Å)	5.251		10.457(12)		5.234(3)
*b*	8.771		15.801(15)		8.751(6)
*c*	15.038	2 0 0	9.584(10)	2 0 0	15.948(15)
α(º)	101.77	−1 0 1	71.87	−2 0 1	103.68(12)
β	89.05	−1 −1 0	115.04	−1 −1 0	69.94(9)
γ	97.57		108.48		95.59(12)
*V*	672.09	Δ = 2	1332.84	Δ = 2	666.58
*SG*	$P\bar{1}$		$P\bar{1}$		$P\bar{1}$
*Z*	2		4		2
*Ref*	Fronckowiak & Hauptman (1976) [32]		Lamotte et al. (1978) [31]		Shin & Jeon (1983) [33]

a The matrix that transforms the Author’s Cell for Structure 2 to the Author’s Cell for Structure 3 is 1 0 0 / 0 1 0 / 1 0 1. As the matrix is characterized by integer elements and a determinant of 1, the two cells describe the same lattice.

b The 3 × 3 matrix in this column transforms the Author’s Cell of Structure 2 to the Author’s Cell of Structure 1.

c The 3 × 3 matrix in this column transforms the Author’s Cell of Structure 3 to the Author’s Cell of Structure 1. The matrices in the Table were determined using NIST*Lattice [18].
